# Assessment of Sexual Behaviour Among HIV-Syphilis Coinfected Individuals From Designated Sexually Transmitted Infection/Reproductive Tract Infection (STI/RTI) Clinics in Mumbai, India

**DOI:** 10.7759/cureus.107920

**Published:** 2026-04-28

**Authors:** Latika Shivkar, Shrikala Acharya, Shwetangi R Shinde, Vijay Kumar Karanjkar, Amol Palkar, Anil Shinde, Madhuri Mhaismale, Maninder S Setia

**Affiliations:** 1 Public Health, Mumbai District AIDS Control Society, Mumbai, IND; 2 Community Medicine, Lokmanya Tilak Municipal Medical College and General Hospital, Mumbai, IND; 3 Data Management, UW International Training and Education Centre for Health Private Limited (I-TECH India), Delhi, IND; 4 Monitoring and Evaluation, Mumbai District AIDS Control Society, Mumbai, IND; 5 Data Monitoring and Documentation, Mumbai District AIDS Control Society, Mumbai, IND; 6 Epidemiology, Mahatma Gandhi Mission Institute of Health Sciences, Navi Mumbai, IND

**Keywords:** coinfection, hiv, hiv and syphilis co-infection, sexual behaviour, syphilis

## Abstract

Introduction: Sexually transmitted infections remain a significant concern among people living with HIV (PLHIV), with syphilis emerging as a common coinfection globally, particularly among men who have sex with men (MSM) and anti-retroviral therapy (ART)-naïve individuals. Understanding the socio-demographic and behavioural characteristics of HIV-syphilis coinfected individuals is important for guiding targeted prevention strategies. This study aimed to examine the associations between socio-demographic characteristics, sexual behaviour patterns, and treatment status among HIV-syphilis coinfected individuals, with a focus on gender differences and MSM versus non-MSM.

Methods: This secondary data analysis included 2,209 PLHIV coinfected with syphilis registered at 27 designated sexually transmitted infections/reproductive tract infection clinics (DSRCs) in Mumbai under the National AIDS and STI Control Programme from April 1, 2022, to March 31, 2025. Socio-demographic, behavioural, and clinical variables were extracted from programme records and analyzed in R, with associations assessed using chi-square or Fisher’s exact tests, as applicable, and multivariable logistic regression was performed, at a significance level of p<0.05. Ethical approval was obtained from the Mumbai District AIDS Control Society, and data were anonymized before analysis.

Results: Among the 2,209 coinfected individuals, 1822 (82.5%) were males, 273 (12.4%) were females, and 114 (5.2%) were transgender individuals. The majority belonged to the 26-35 year age group (n = 767, 34.7%). Significant differences were observed across gender groups in education, marital status, sexual practices, partner types, condom use, and treatment status (p < 0.05). Vaginal intercourse was the most common sexual act overall (n = 1434, 76.6%), while anal and oral sexual practices were more frequently reported among males and transgender individuals (p < 0.001). Spousal partners were the most commonly reported type of partner (n = 886, 51.0%), followed by commercial (n = 808, 45.9%) and casual partners (n = 794, 44.3%). Consistent condom use was highest with casual partners (n = 377, 49.2%) but lower with regular partners (n = 71, 30.6%). Among males, 398 (21.8%) identified as MSM, who were significantly younger, more educated, and more likely to report anal sex and casual partners (p < 0.05). Overall, 60.3% (n = 1332) of participants were ART-experienced. In multivariable analysis, MSM status was independently associated with unmarried status, higher education, and anal intercourse. Increasing age, male and transgender identity were associated with higher odds of being ART-experienced, while engagement in commercial partnerships and anal intercourse were associated with lower odds.

Conclusion: HIV-syphilis coinfection in our study appears to be sustained through overlapping sexual networks involving casual, commercial, and marital partnerships combined with inconsistent condom use. Strengthened screening for syphilis, targeted prevention interventions, and behavioural counselling for risk reduction among PLHIV are essential to reduce ongoing transmission.

## Introduction

Globally, sexually transmitted infections (STIs) remain a persistent concern among people living with HIV (PLHIV), with syphilis emerging as a significant coinfection with rising prevalence across diverse settings [1-3]. In 2022, a meta-analysis among 34,740 PLHIV in China reported a pooled syphilis prevalence of 19.9% (95% CI: 15.4-24.8%) [4]. Hospital-based cohorts have documented prevalence rates ranging from 7-25% in PLHIV [5-7], with rates exceeding 40% being reported in anti-retroviral therapy (ART)-naïve individuals [8].

The biological interaction between HIV and syphilis is bidirectional and synergistic. Syphilitic lesions disrupt mucosal integrity and increase local recruitment of CD4 lymphocyte cells, thereby facilitating HIV acquisition and transmission [9,10]. On the other hand, HIV alters the clinical course of syphilis, leading to atypical manifestations, increased rates of neurosyphilis, ophthalmic complications, and higher rates of treatment failure [9,11,12]. Coinfection has been associated with elevated HIV viral loads, reduced CD4 lymphocyte counts, impaired immune recovery, and a higher risk of virological failure despite ART [8,13-15].

Epidemiologically, the cohort of men who have sex with men (MSM) consistently shows a strong relationship with HIV-syphilis coinfection [3,11,16]. Surveillance data from developed countries reveal rising male-to-female ratios of syphilis notifications over time, largely driven by MSM transmission networks [17]. Certain behavioural mechanisms have been highlighted as contributing to this resurgence. Anal intercourse carries a higher transmission risk due to epithelial abrasions and the highly vascular rectal mucosa, facilitating direct bloodstream access [2,12]. Regarding patient age, few studies reported a higher prevalence among older PLHIV [1,4,11], probably due to cumulative exposure, whereas other studies identified increased risk in the 25-34 year age group due to higher sexual activity and unsafe sexual practices [10,18].

The expansion of digital networking platforms and mobile dating applications has further increased partner turnover and facilitated condomless encounters [12]. In parallel, improved quality of life due to ART, perceptions associated with "undetectable = untransmittable (U=U)," and "safe sex fatigue" have been linked to unprotected sexual intercourse among PLHIV [12,19]. Substance use, including recreational drug use and transactional sex for drug exchange, further augments STI vulnerability [1,12,20]. Studies consistently demonstrate that multiple partners, early sexual debut, receptive anal intercourse, inconsistent condom use, and drug use independently increase the risk of HIV-syphilis coinfection [1,2,10].

In India, the National AIDS and STD Control Programme (NACP) introduced syphilis screening among STI clients in its early phases, and in 2015, this was extended to cover universal screening of all pregnant women [21]. Under Phase V, dual HIV-syphilis testing is encouraged for clients attending integrated counselling and testing centres (ICTCs) and designated STI/RTI clinics (DSRCs) [22,23]. However, there is limited evidence from India examining sexual behaviour patterns among HIV-syphilis coinfected individuals using large-scale programmatic data. Analysis of routinely collected programme data can provide valuable real-world insights to guide targeted prevention strategies within public health systems. Given the rising global burden of HIV-syphilis coinfection, its impact on immunological and treatment outcomes, and the evolving sexual practices among PLHIV, the present study was undertaken to analyse the association between socio-demographic factors, sexual behaviour patterns, and treatment status with gender among HIV-syphilis coinfected individuals. It further aimed to examine these associations across male typologies, specifically comparing MSM and non-MSM. Additionally, the study aimed to assess the relationship between treatment status and selected socio-demographic factors and sexual behaviour patterns within the study population.

## Materials and methods

Study setting and design

The study was conducted in Mumbai, Maharashtra, India, at 27 DSRCs that provide services under the National AIDS and STD Control Programme (NACP). These clinics provide screening, diagnosis, and management of STIs, including syphilis.

The present study was a cross-sectional analytical study using the extraction and analysis of secondary data generated through routine programme activities under the NACP for the period April 1, 2022, to March 31, 2025.

Study population and sampling

The study population comprised adult HIV-syphilis coinfected individuals who were registered for care at the 27 DSRCs in Mumbai during the study period. A total of 2,209 HIV-syphilis coinfected clients were identified and included in the analysis. Since all eligible coinfected clients during the defined time frame were included, complete enumeration was performed, and no sampling technique was applied.

Variables and measurement

Socio-demographic variables, including age, gender, education, and marital status, were extracted for analysis. Additionally, sexual behaviour variables were extracted, including the type of sexual partner (casual, commercial, regular, or spouse), type of sexual act (vaginal, anal, or oral), and condom use (categorised as always, frequently, rarely, or never), during a recall period of three months, which was chosen for the study.

Partner types in this study were defined in alignment with the National Integrated Biological and Behavioural Surveillance (IBBS) 2014-15 definitions [24]. A regular partner refers to a person with whom the client has an ongoing, stable sexual relationship characterised by emotional attachment and repeated sexual contact over time, but who is not legally married (e.g., boyfriend/girlfriend or live-in partner). A spouse is a legally married partner within a formal marital relationship. A casual partner denotes a non-regular sexual partner with whom sexual contact occurs occasionally or intermittently without emotional commitment or a long-term relationship (e.g., one-time or short-term partners). A commercial partner refers to a sexual partner with whom sexual activity occurs in exchange for money, goods, or other benefits, encompassing transactional sex, including sex workers or their clients [24].

Regarding consistent condom use, the IBBS defines it as condom use during every sexual act in the past month. As the present study relied on programmatic data with categorical responses, clients reporting condom use as “always” or “frequently” were operationally classified as having consistent condom use [24].

Clinical variables included the duration of ART. PLHIV who had been on ART for less than six months at the time of syphilis diagnosis were categorised as “Treatment-naïve”, whereas those on ART for more than 6 months were categorised as “ART-experienced”. This is consistent with previous studies, where a duration of less than six months on ART is often considered indicative of newly detected coinfection, whereas longer ART duration represents established engagement in HIV care [6,13].

Data collection and statistical analysis

Data were extracted from client cards maintained at DSRCs for socio-demographic and behavioural variables, and from the Master Line List at ART centres for ART initiation and duration details. The dataset was anonymised before analysis.

The anonymised dataset was entered and analysed using R version 4.5.2 (R Foundation for Statistical Computing, Vienna, Austria). Continuous variables, including age and duration of ART, were converted into categorical variables for analysis. Descriptive statistics were used to summarise the data. Associations between categorical variables were assessed using the chi-square or Fisher’s test as applicable, and a p-value of less than 0.05 was considered statistically significant.

Missing data were observed for sexual behaviour variables, including the type of sexual act and type of partner. These were handled by creating a new category labelled “No response” to retain observations in the analysis. Multivariable logistic regression analysis was performed to identify factors independently associated with the outcomes of interest (MSM vs non-MSM status and treatment status). Variables with a p-value <0.20 in bivariate analysis, along with those of epidemiological relevance, were included in the regression models. Adjusted odds ratios (AOR) with 95% confidence intervals (CI) were reported. Model fit was assessed using Akaike Information Criterion (AIC) and Bayesian Information Criterion (BIC), with lower values indicating better model fit.

Data quality, completeness, and validation

Since the study utilised secondary programmatic data, measures were undertaken to ensure data quality and completeness, including cross-verification with source registers, routine data validation checks at the facility and supervisory levels, and exclusion of records with missing or inconsistent key variables; the data extracted were cleaned, coded, and validated before analysis.

Ethical considerations

Ethical approval for the study was obtained from the Institutional Ethics Committee of the Mumbai District AIDS Control Society (MDACS); Ref No. MDACS-IRB/002/2025 dated 25th October, 2025. As the study involved secondary analysis of routinely collected programme data, a waiver of informed consent was granted. Strict confidentiality was maintained throughout the study. All personal identifiers were removed before analysis to ensure data anonymity.

## Results

A total of 2,209 HIV-syphilis coinfected individuals were included in the analysis. Of these, 1822 (82.5%) were men, 273 (12.4%) were women, and 114 (5.2%) were transgender individuals. A total of 877 (39.7%) were ART-naïve, and 1,332 (60.3%) were ART-experienced. The median ART duration among ART-experienced individuals was 75 months (IQR: 34.2-123.0).

Table 1 shows the socio-demographic and behavioural characteristics of HIV-syphilis coinfected individuals stratified by gender. The largest proportion was in the 26-35 year age group (n = 767, 34.7%), followed by 36-45 years (n = 527, 23.9%), with a statistically significant difference observed across genders (p < 0.001). Among men and transgender individuals, the largest proportion was observed in the 26-35 years age group (n = 647, 35.5% and n = 48, 42.1%, respectively), whereas among women, the highest proportion was in the 36-45 years age group (n = 78, 28.6%). Regarding education, secondary school (n = 873, 39.5%) was the most commonly attained level, followed by primary school (n = 453, 20.5%). Illiteracy was significantly higher among transgender individuals (n = 37, 32.5%) and women (n = 70, 25.6%) compared to men (n = 78, 4.3%) (p <0.001). Regarding marital status, most of the participants were either unmarried (n = 990, 44.8%) or married individuals (n = 901, 40.8%). Marital status showed a statistically significant association with gender (p < 0.001), with a larger proportion of unmarried men (n = 866, 47.5%) as compared to women (n = 17, 6.2%). About 93.9% (n = 107) of transgender individuals were unmarried.

**Table 1:** Socio-demographic and behavioural characteristics of HIV-syphilis coinfected individuals stratified by their gender †Participants could report more than one type of sexual partner and type of sexual act; therefore, the frequencies exceed the total sample size, and a cumulative total cannot be calculated. Each category was analysed separately using the chi-square test. *chi-square test; **Fisher’s exact test

Variable	Male (N = 1822); n (%)	Female (N = 273); n (%)	Transgender (N = 114); n (%)	Total (N = 2209); n (%)	Test	p-value
Age group						
18-25 years	285 (15.6%)	17 (6.2%)	23 (20.2%)	325 (14.7%)	^𝛘^^2^ = 53.5, df = 8	<0.001*
26-35 years	647 (35.5%)	72 (26.4%)	48 (42.1%)	767 (34.7%)		
36-45 years	427 (23.4%)	78 (28.6%)	22 (19.3%)	527 (23.9%)		
46-55 years	300 (16.5%)	66 (24.2%)	20 (17.5%)	386 (17.5%)		
≥ 56 years	163 (8.9%)	40 (14.7%)	1 (0.9%)	204 (9.2%)		
Total	1822 (82.5%)	273 (12.4%)	114 (5.2%)	2209 (100.0%)		
Education						
Illiterate	78 (4.3%)	70 (25.6%)	37 (32.5%)	185 (8.4%)	^𝛘^^2^ = 327, df = 8	<0.001*
Primary school	330 (18.1%)	97 (35.5%)	26 (22.8%)	453 (20.5%)		
Secondary school	760 (41.7%)	81 (29.7%)	32 (28.1%)	873 (39.5%)		
Higher secondary	372 (20.4%)	11 (4.0%)	16 (14.0%)	399 (18.1%)		
Graduate and above	282 (15.5%)	14 (5.1%)	3 (2.6%)	299 (13.5%)		
Total	1822 (82.5%)	273 (12.4%)	114 (5.2%)	2209 (100.0%)		
Marital status						
Unmarried/single	866 (47.5%)	17 (6.2%)	107 (93.9%)	990 (44.8%)	^𝛘^^2^ = 463.6, df = 8	<0.001*
Married	762 (41.8%)	132 (48.4%)	7 (6.1%)	901 (40.8%)		
Separated/divorced	102 (5.6%)	36 (13.2%)	0	138 (6.2%)		
Widow/widower	92 (5.0%)	88 (32.2%)	0	180 (8.1%)		
Total	1822 (82.5%)	273 (12.4%)	114 (5.2%)	2209 (100.0%)		
Type of sexual act†						
Vaginal	1207 (77.2%)	223 (97.0%)	4 (5.1%)	1434 (76.6%)	^𝛘^^2^ = 275.8, df = 2	<0.001*
Anal	481 (34.1%)	4 (2.1%)	94 (91.3%)	579 (33.9%)	^𝛘^^2^ = 239.1, df = 2	<0.001*
Oral	346 (25.7%)	9 (4.8%)	49 (62.8%)	404 (25.1%)	^𝛘^^2^ = 100.6, df = 2	<0.001*
Type of partner†						
Regular partner	210 (15.4%)	12 (5.9%)	16 (20.3%)	238 (14.5%)	^𝛘^^2^ = 15.3, df = 2	<0.001*
Casual partner	717 (47.8%)	28 (13.6%)	49 (57.6%)	794 (44.3%)	^𝛘^^2^ = 92.2, df = 2	<0.001*
Commercial partner	687 (47.1%)	58 (28.4%)	63 (63.6%)	808 (45.9%)	^𝛘^^2^ = 38.4, df = 2	<0.001*
Spouse	731 (51.3%)	152 (64.7%)	3 (3.9%)	886 (51.0%)	^𝛘^^2^ = 86.0, df = 2	<0.001*
Consistent condom use†						
Regular partner	62 (30.0%)	4 (44.44%)	5 (31.2%)	71 (30.6%)	Fisher’s Exact test	0.68**
Casual partner	337 (48.5%)	11 (42.3%)	29 (64.4%)	377 (49.2%)	^𝛘^^2^ = 4.8, df = 2	0.09*
Commercial partner	247 (36.9%)	39 (73.6%)	24 (38.7%)	310 (39.5%)	^𝛘^^2^ = 27.7, df = 2	<0.001*
Spouse	285 (41.7%)	37 (28.9%)	0	322 (39.6%)	Fisher’s Exact test	0.008**
Treatment status						
Treatment-naïve	737 (40.5%)	109 (39.9%)	31 (27.2%)	877 (39.7%)	^𝛘^^2^ = 7.9, df = 2	0.02*
ART-experienced	1085 (59.5%)	164 (60.1%)	83 (72.8%)	1332 (60.3%)		
Total	1822 (82.5%)	273 (12.4%)	114 (5.2%)	2209 (100.0%)		

Regarding sexual behaviour, significant differences in behavioural patterns across gender groups were observed for type of sexual act, type of partner, and consistent condom use (p < 0.05). Vaginal intercourse was the most commonly reported sexual act (n = 1434, 76.6%), predominantly reported among women (n = 223, 97.0%) and men (n = 1207, 77.2%). Overall, anal intercourse (n = 579, 33.9%) and oral sex (n = 404, 25.1%) were less common, but anal intercourse was reported mainly among transgender individuals (n = 94, 91.3%). In terms of partner type, spousal partners were reported by 51.0% (n = 886) of participants, followed by commercial partners (n = 808, 45.9%), casual partners (n = 794, 44.3%), and regular partners (n = 238, 14.5%). Casual and commercial partners were more frequently reported among men and transgender individuals, whereas spousal partners were most commonly reported among women (p < 0.001). Consistent condom use varied by partner type, with 49.2% (n = 377) reporting consistent use with casual partners, 39.5% (n = 310) with commercial partners, 39.6% (n = 322) with spouses, and 30.6% (n = 71) with regular partners. Consistent condom use with regular partners was fairly similar across all genders (p = 0.68). With casual partners, consistent condom use was highest among transgender individuals (n = 29, 64.4%), followed by men (n = 337, 48.5%) and women (n = 11, 42.3%) (p = 0.09). Among those reporting commercial partners, consistent condom use was significantly higher among women (n = 39, 73.6%), compared to transgender individuals (n = 24, 38.7%) and men (n = 247, 36.9%) (p < 0.001). With spousal partners, consistent condom use was reported by 41.7% (n = 285) of men as compared to 28.9% (n = 37) of women (p = 0.008).

Regarding duration of ART, a similar pattern was observed across men and women, 40.5% (n = 737) and 39.9% (n = 109) were treatment-naïve, respectively. However, among transgender individuals, there was a higher proportion of ART-experienced individuals (n = 83, 72.8%) (p = 0.02).

Table 2 presents the socio-demographic and behavioural characteristics of HIV-syphilis coinfected men stratified by typology: MSM and non-MSM. Among the 1,822 coinfected men, 21.8% (n = 398) were MSM and 78.2% (n = 1424) were non-MSM. A significantly higher proportion of MSM belonged to the younger age groups, particularly 26-35 years (n = 186, 46.7%) and 18-25 years (n = 105, 26.4%), compared to non-MSM, where 26-35 years (n = 461, 32.4%) and 36-45 years (n = 350, 24.6%) were most common (p < 0.001). With respect to educational status, MSM were generally more educated than non-MSM (p < 0.001). A higher proportion of MSM had completed secondary school (n = 130, 32.7%) and graduate level or above (n = 121, 30.4%), compared to non-MSM. Regarding marital status, the majority of MSM were unmarried or single (n = 330, 82.9%), whereas marriage was more common among non-MSM (n = 712, 50.0%) (p < 0.001).

**Table 2:** Socio-demographic and behavioural characteristics of HIV-syphilis coinfected men stratified by typology †Participants could report more than one type of sexual partner and type of sexual act; therefore, the frequencies exceed the total sample size, and a cumulative total cannot be calculated. Each category was analysed separately using the chi-square test. MSM: Men who have sex with men

Variable	MSM (N = 398); n (%)	Non-MSM (N = 1424); n (%)	Total (N = 1822); n (%)	Chi-square test statistic	p-value
Age group					
18-25 years	105 (26.4%)	180 (12.6%)	285 (15.6%)	^𝛘^^2^ = 123.4, df = 4	<0.001
26-35 years	186 (46.7%)	461 (32.4%)	647 (35.5%)		
36-45 years	77 (19.3%)	350 (24.6%)	427 (23.4%)		
46-55 years	21 (5.3%)	279 (19.6%)	300 (16.5%)		
≥ 56 years	9 (2.3%)	154 (10.8%)	163 (8.9%)		
Total	398 (21.8%)	1424 (78.2%)	1822 (100.0%)		
Education					
Illiterate	9 (2.3%)	69 (4.8%)	78 (4.3%)	^𝛘^^2^ = 140.7, df = 4	<0.001
Primary school	26 (6.5%)	304 (21.3%)	330 (18.1%)		
Secondary school	130 (32.7%)	630 (44.2%)	760 (41.7%)		
Higher secondary	112 (28.1%)	260 (18.3%)	372 (20.4%)		
Graduate and above	121 (30.4%)	161 (11.3%)	282 (15.5%)		
Total	398 (21.8%)	1424 (78.2%)	1822 (100.0%)		
Marital status					
Unmarried/single	330 (82.9%)	536 (37.6%)	866 (47.5%)	^𝛘^^2^ = 257.1, df = 3	<0.001
Married	50 (12.6%)	712 (50.0%)	762 (41.8%)		
Separated/divorced	12 (3.0%)	90 (6.3%)	102 (5.6%)		
Widow/widower	6 (1.5%)	86 (6.0%)	92 (5.1%)		
Total	398 (21.8%)	1424 (78.2%)	1822 (100.0%)		
Type of sexual act†					
Vaginal	88 (29.0%)	1119 (88.7%)	1207 (77.2%)	^𝛘^^2^ = 490.8, df = 1	<0.001
Anal	304 (89.4%)	177 (16.5%)	481 (34.1%)	^𝛘^^2^ = 607.5, df = 1	<0.001
Oral	202 (69.4%)	144 (13.7%)	346 (25.7%)	^𝛘^^2^ = 368.1, df = 1	<0.001
Type of partner†					
Regular partner	123 (41.1%)	87 (8.2%)	210 (15.4%)	^𝛘^^2^ = 192.3, df = 1	<0.001
Casual partner	289 (85.5%)	428 (36.8%)	717 (47.8%)	^𝛘^^2^ = 247.0, df = 1	<0.001
Commercial partner	86 (29.2%)	601 (51.6%)	687 (47.1%)	^𝛘^^2^ = 46.8, df = 1	<0.001
Spouse	84 (28.9%)	647 (57.1%)	731 (51.3%)	^𝛘^^2^ = 72.8, df = 1	<0.001
Consistent condom use†					
Regular partner	43 (35.2%)	19 (22.4%)	62 (30.0%)	^𝛘^^2^ = 3.4, df = 1	0.07
Casual partner	114 (40.1%)	217 (52.8%)	331 (47.6%)	^𝛘^^2^ = 10.3, df = 1	0.001
Commercial partner	31 (37.8%)	266 (45.7%)	297 (44.7%)	^𝛘^^2^ = 1.5, df = 1	0.22
Spouse	26 (31.7%)	259 (43.1%)	285 (41.7%)	^𝛘^^2^ = 3.4, df = 1	0.07
Treatment status					
Treatment-naïve	195 (49.0%)	542 (38.1%)	737 (40.4%)	^𝛘^^2^ = 15.0, df = 1	<0.001
ART-experienced	203 (51.0%)	882 (61.9%)	1085 (59.6%)		
Total	398 (21.8%)	1424 (78.2%)	1822 (100.0%)		

Behavioural characteristics, such as the type of sexual act and type of sexual partners, differed significantly by typology (p < 0.001 each). Anal intercourse was predominantly reported among MSM (n = 304, 89.4%), whereas vaginal intercourse was primarily reported among non-MSM (n = 1119, 88.7%). Oral sex was also more frequently reported among MSM (n = 202, 69.4%) compared to non-MSM (n = 144, 13.7%). Casual partners were reported more frequently among MSM (n = 289, 85.5%) as compared to non-MSM (n = 428, 36.8%), whereas commercial partners (n = 601, 51.6%) and spouses (n = 647, 57.1%) were more commonly reported among non-MSM. Regarding consistent condom use, a statistically significant difference was observed only for casual partners, where MSM reported lower consistent condom use (n = 114, 40.1%) compared to non-MSM (n = 217, 52.8%) (p = 0.001). Condom use with regular partners (p = 0.07), commercial partners (p = 0.22), and spouses (p = 0.07) did not differ significantly between MSM and non-MSM. Additionally, regarding treatment status, a significantly higher proportion of MSM were treatment-naïve (n = 195, 49.0%) compared to non-MSM (n = 542, 38.1%) (p < 0.001).

Table 3 presents the socio-demographic and behavioural characteristics of HIV-syphilis coinfected individuals stratified by treatment duration. Among the 2,209 coinfected individuals, 877 (39.7%) were treatment-naïve, while 1,332 (60.3%) were ART-experienced.

**Table 3:** Socio-demographic and behavioural characteristics of HIV-syphilis coinfected individuals stratified by treatment duration †Participants could report more than one type of sexual partner and type of sexual act; therefore, the frequencies exceed the total sample size, and a cumulative total cannot be calculated. Each category was analysed separately using the chi-square test. ART: anti-retroviral therapy

Variable	Treatment-naïve (N = 877); n (%)	ART-experienced (N = 1332); n (%)	Total (N = 2209); n (%)	Chi-square test statistic	p-value
Age group					
18-25 years	217 (24.7%)	108 (8.1%)	325 (14.7%)	^𝛘^^2^ = 181.7, df = 4	<0.001
26-35 years	341 (38.9%)	426 (32.0%)	767 (34.7%)		
36-45 years	182 (20.8%)	345 (25.9%)	527 (23.9%)		
46-55 years	98 (11.2%)	288 (21.6%)	386 (17.5%)		
≥ 56 years	39 (4.4%)	165 (12.4%)	204 (9.2%)		
Total	877 (39.7%)	1332 (60.3%)	2209 (100.0%)		
Gender					
Male	737 (84.0%)	1085 (81.5%)	1822 (82.5%)	^𝛘^^2^ = 7.9, df = 2	0.02
Female	109 (12.4%)	164 (12.3%)	273 (12.4%)		
Transgender	31 (3.5%)	83 (6.2%)	114 (5.2%)		
Total	877 (39.7%)	1332 (60.3%)	2209 (100.0%)		
Education					
Illiterate	59 (6.7%)	126 (9.5%)	185 (8.4%)	^𝛘^^2^ = 27.7, df = 4	<0.001
Primary school	166 (18.9%)	287 (21.5%)	453 (20.5%)		
Secondary school	323 (36.8%)	550 (41.3%)	873 (39.5%)		
Higher secondary	178 (20.3%)	221 (16.6%)	399 (18.1%)		
Graduate and above	151 (17.2%)	148 (11.1%)	299 (13.5%)		
Total	877 (39.7%)	1332 (60.3%)	2209 (100.0%)		
Marital status					
Unmarried/single	458 (52.2%)	532 (39.9%)	990 (44.8%)	^𝛘^^2^ = 45.5, df = 3	<0.001
Married	334 (38.1%)	567 (42.6%)	901 (40.8%)		
Separated/divorced	44 (5.0%)	94 (7.1%)	138 (6.3%)		
Widow/widower	41 (4.7%)	139 (10.4%)	180 (8.2%)		
Total	877 (39.7%)	1332 (60.3%)	2209 (100.0%)		
Type of sexual act†					
Vaginal	571 (73.5%)	863 (78.8%)	1434 (76.6%)	^𝛘^^2^ = 6.9, df = 1	0.009
Anal	294 (40.4%)	285 (29.1%)	579 (33.9%)	^𝛘^^2^ = 23.5, df = 1	<0.001
Oral	229 (33.5%)	175 (18.9%)	404 (25.1%)	^𝛘^^2^ = 44.3, df = 1	<0.001
Type of partner†					
Regular partner	123 (18.2%)	115 (11.9%)	238 (14.5%)	^𝛘^^2^ = 12.4, df = 1	<0.001
Casual partner	375 (48.9%)	419 (40.9%)	794 (44.3%)	^𝛘^^2^ = 11.1, df = 1	<0.001
Commercial partner	351 (50.1%)	457 (43.1%)	808 (45.9%)	^𝛘^^2^ = 8.1, df = 1	0.005
Spouse	335 (47.1%)	551 (53.8%)	886 (51.0%)	^𝛘^^2^ = 7.4, df = 1	0.006
Consistent condom use†					
Regular partner	37 (31.4%)	34 (29.8%)	71 (30.6%)	^𝛘^^2^ = 0.0, df = 1	0.91
Casual partner	165 (45.0%)	212 (53.1%)	377 (49.2%)	^𝛘^^2^ = 4.8, df = 1	0.03
Commercial partner	82 (24.6%)	228 (50.6%)	310 (39.5%)	^𝛘^^2^ = 53, df = 1	<0.001
Spouse	74 (23.3%)	248 (50.0%)	322 (39.6%)	^𝛘^^2^ = 56.3, df = 1	<0.001

A higher proportion of treatment-naïve individuals belonged to the younger age groups, particularly 18-25 years (n = 217, 24.7%) and 26-35 years (n = 341, 38.9%), whereas ART-experienced individuals were more commonly represented in older age groups, including 36-45 years (n = 345, 25.9%) and 46-55 years (n = 288, 21.6%) (p < 0.001). With respect to gender, men constituted the majority in both groups, accounting for 84.0% (n = 737) of treatment-naïve and 81.5% (n = 1085) of ART-experienced individuals, with a small but statistically significant difference across gender categories (p = 0.02). Educational status also differed significantly between groups (p < 0.001), with higher secondary and graduate levels more common among treatment-naïve individuals. Regarding marital status, unmarried individuals were more frequently treatment-naïve (n = 458, 52.2%), while married (n = 567, 42.6%) and widowed individuals (n = 139, 10.4%) were more commonly ART-experienced, showing a statistically significant association (p < 0.001).

Behavioural characteristics also differed significantly by treatment duration. Vaginal intercourse was more frequently reported among ART-experienced individuals (n = 863, 78.8%), whereas anal (n = 294, 40.4%) and oral sexual practices (n = 229, 33.5%) were more commonly reported among treatment-naïve individuals. In terms of type of sexual partner, treatment-naïve individuals more frequently reported regular partners (n = 123, 18.2%), casual partners (n = 375, 48.9%), and commercial partners (n = 351, 50.1%). In contrast, spousal partners were more commonly reported among ART-experienced individuals (n = 551, 53.8%). Regarding patterns of consistent condom use, ART-experienced individuals reported higher consistent condom use with casual partners (n = 212, 53.1%), commercial partners (n = 228, 50.6%), and spouses (n = 248, 50.0%) compared to treatment-naïve individuals, while condom use with regular partners showed only marginal differences between the groups.

Table 4 presents the univariate and multivariate logistic regression analysis of sociodemographic and sexual behavioural factors associated with MSM status. In univariate analysis, younger age, unmarried status, higher education, presence of regular and casual partners, engagement in anal intercourse, and treatment-naïve status were significantly associated with MSM status (p < 0.001). However, after adjustment, age, commercial partner status, and treatment status were no longer significant predictors. Unmarried individuals had significantly higher odds of being MSM (AOR: 2.69; 95% CI: 1.70-4.30), and those with graduation or higher education also demonstrated increased odds (AOR: 2.15; 95% CI: 1.20-3.91). Behavioural factors remained strongly associated, with individuals reporting regular partners (AOR: 5.00; 95% CI: 3.12-8.10) and casual partners (AOR: 6.81; 95% CI: 4.24-11.17) showing significantly higher odds of MSM status. Anal intercourse emerged as the strongest factor (AOR: 12.71; 95% CI: 7.79-21.15), while vaginal intercourse was inversely associated (AOR: 0.41; 95% CI: 0.26-0.65). The non-response for casual partner (AOR: 9.46; 95% CI: 4.47-20.63) and vaginal intercourse (AOR: 3.54; 95% CI: 1.89-6.88) were associated with higher odds of MSM status, suggesting potential differential disclosure patterns. The model demonstrated good fit (AIC = 1010.93; BIC = 1110.07).

**Table 4:** Univariate and multivariate regression analysis of sociodemographic and sexual behavioural factors associated with MSM status Model fit statistics: AIC = 1010.93, BIC = 1110.07; MSM: Men who have sex with men, ART: Antiretroviral therapy

Variable	Category	Crude OR (95% CI)	p-value	Adjusted OR (95% CI)	p-value
Age group	18-35 years	1.0 (Ref)	-	1.0 (Ref)	-
	36-55 years	0.34 (0.27-0.44)	<0.001	1.07 (0.72-1.60)	0.73
	≥56 years	0.13 (0.06-0.24)	<0.001	0.44 (0.16-1.10)	0.09
Marital status	Married	1.0 (Ref)	-	1.0 (Ref)	-
	Unmarried/single	8.77 (6.44-12.17)	<0.001	2.69 (1.70-4.30)	<0.001
	Currently not with spouse (separated/ divorced/ widowed)	1.46 (0.81-2.51)	0.19	1.12 (0.54-2.24)	0.76
Education	Illiterate or primary school	1.0 (Ref)	-	1.0 (Ref)	-
	Secondary or higher secondary	2.90 (2.02-4.28)	<0.001	1 (0.60-1.68)	0.99
	Graduation and above	8.01 (5.32-12.33)	<0.001	2.15 (1.20-3.91)	0.01
Regular partner	No	1.0 (Ref)	-	1.0 (Ref)	-
	Yes	7.86 (5.73-10.83)	<0.001	5.00 (3.12-8.10)	<0.001
	No response	1.53 (1.16-2.01)	0.002	0.35 (0.16-0.77)	0.01
Casual partner	No	1.0 (Ref)	-	1.0 (Ref)	-
	Yes	10.13 (7.38-14.17)	<0.001	6.81 (4.24-11.17)	<0.001
	No response	3.45 (2.31-5.18)	<0.001	9.46 (4.47-20.63)	<0.001
Commercial partner	No	1.0 (Ref)	-	1.0 (Ref)	-
	Yes	0.39 (0.29-0.51)	<0.001	0.82 (0.52-1.28)	0.38
	No response	1.07 (0.81-1.41)	0.65	0.72 (0.33-1.59)	0.41
Vaginal intercourse	No	1.0 (Ref)	-	1.0 (Ref)	-
	Yes	0.05 (0.04-0.07)	<0.001	0.41 (0.26-0.65)	<0.001
	No response	0.39 (0.28-0.53)	<0.001	3.54 (1.89-6.88)	<0.001
Anal intercourse	No	1.0 (Ref)	-	1.0 (Ref)	-
	Yes	42.70 (29.53-63.42)	<0.001	12.71 (7.79-21.15)	<0.001
	No response	4.10 (2.67 to 6.37)	<0.001	1.45 (0.68-3.01)	0.33
Treatment status	ART-experienced	1.0 (Ref)	-	1.0 (Ref)	-
	Treatment-naïve	1.56 (1.25-1.96)	<0.001	0.90 (0.64-1.26)	0.53

Table 5 presents the univariate and multivariate regression analysis of sociodemographic and sexual behavioural factors associated with treatment status. In univariate analysis, increasing age and transgender identity were associated with higher odds of being ART-experienced, whereas unmarried status and behavioural factors, including regular, casual, and commercial partners, as well as anal intercourse, were associated with lower odds. In multivariate analysis, age remained a strong independent factor, with individuals aged 36-55 years (AOR: 2.21; 95% CI: 1.79-2.73) and ≥56 years (AOR: 3.86; 95% CI: 2.61-5.82) showing significantly higher odds of being ART-experienced (p < 0.001). Men (AOR: 1.67; 95% CI: 1.22-2.27) and transgender individuals (AOR: 4.29; 95% CI: 2.43-7.67) had higher odds compared to women. Individuals not currently living with a spouse were also more likely to be ART-experienced (AOR: 1.50; 95% CI: 1.11-2.05). Among behavioural factors, reporting a commercial partner (AOR: 0.56; 95% CI: 0.45-0.70) and engaging in anal intercourse (AOR: 0.61; 95% CI: 0.44-0.84) were independently associated with lower odds of being ART-experienced. Other variables, including education, regular and casual partner status, and vaginal intercourse, were not significant after adjustment. Among the “No response” categories, non-response for casual partner remained significantly associated with higher odds (AOR: 2.28; 95% CI: 1.49-3.50), while non-response for commercial partner was associated with lower odds (AOR: 0.50; 95% CI: 0.33-0.76). The model demonstrated acceptable fit (AIC = 2760.38; BIC = 2874.39).

**Table 5:** Univariate and multivariate regression analysis of sociodemographic and sexual behavioural factors associated with treatment status Model fit statistics: AIC = 2760.38, BIC = 2874.39

Variable	Category	Crude OR (95% CI)	p-value	Adjusted OR (95% CI)	p-value
Age group	18-35 years	1.0 (Ref)	-	1.0 (Ref)	-
	36-55 years	2.36 (1.96-2.84)	<0.001	2.21 (1.79-2.73)	<0.001
	≥56 years	4.44 (3.10-6.49)	<0.001	3.86 (2.61-5.82)	<0.001
Gender	Female	1.0 (Ref)	-	1.0 (Ref)	-
	Male	0.97 (0.75-1.26)	0.84	1.67 (1.22-2.27)	<0.001
	Transgender	1.78 (1.11-2.90)	0.02	4.29 (2.43-7.67)	<0.001
Marital status	Married	1.0 (Ref)	-	1.0 (Ref)	-
	Unmarried/ single	0.68 (0.57-0.82)	<0.001	1.04 (0.82-1.33)	0.73
	Currently not with spouse (separated/ divorced/ widowed)	1.56 (1.18-2.08)	0.002	1.50 (1.11-2.05)	0.01
Education	Illiterate or primary school	1.0 (Ref)	-	1.0 (Ref)	-
	Secondary or higher secondary	0.85 (0.70-1.03)	0.11	1.12 (0.89 to 1.40)	0.35
	Graduation and above	0.54 (0.41-0.71)	<0.001	0.90 (0.65-1.24)	0.50
	Missing	1.29 (0.36-6.03)	0.72	1.16 (0.29-5.77)	0.84
Regular partner	No	1.0 (Ref)	-	1.0 (Ref)	-
	Yes	0.61 (0.46-0.80)	<0.001	0.78 (0.57-1.07)	0.12
	No response	1.18 (0.96-1.45)	0.11	0.90 (0.57-1.44)	0.66
Casual partner	No	1.0 (Ref)	-	1.0 (Ref)	-
	Yes	0.73 (0.60-0.88)	0.001	0.92 (0.73-1.18)	0.51
	No response	1.82 (1.42-2.35)	<0.001	2.28 (1.49-3.50)	<0.001
Commercial partner	No	1.0 (Ref)	-	1.0 (Ref)	-
	Yes	0.76 (0.63-0.92)	0.005	0.56 (0.45-0.70)	<0.001
	No response	0.90 (0.72-1.14)	0.37	0.50 (0.33-0.76)	0.001
Vaginal intercourse	No	1.0 (Ref)	-	1.0 (Ref)	-
	Yes	1.33 (1.08-1.66)	0.009	0.77 (0.55-1.07)	0.12
	No response	2.10 (1.56-2.85)	<0.001	1.03 (0.68-1.55)	0.91
Anal intercourse	No	1.0 (Ref)	-	1.0 (Ref)	-
	Yes	0.61 (0.50-0.75)	<0.001	0.61 (0.44-0.84)	0.002
	No response	1.48 (1.18-1.86)	<0.001	1.12 (0.80-1.55)	0.513

Overall, HIV-syphilis coinfection in this cohort was predominantly observed among men in the sexually active 26-35 year age group and was associated with distinct behavioural patterns across gender, male typology, and ART treatment status, particularly with respect to sexual practices, partner types, and condom use.

## Discussion

The present study assessed the socio-demographic characteristics, sexual behaviour patterns, and treatment status of 2,209 HIV-syphilis coinfected individuals registered at 27 DSRCs in Mumbai.

Age distribution in the present study showed that the largest proportion of coinfected individuals was in the younger age groups, a finding consistent with previous research that highlights sexually active young adults as a key population affected by HIV-syphilis coinfection, possibly due to multiple partners, inconsistent condom use, and risk-taking sexual behaviour [10]. At the same time, multivariable analysis demonstrated that ART-experienced individuals were more likely to be older, suggesting that cumulative exposure, prolonged survival on ART, and continued engagement in sexual networks contribute to the persistence of coinfection among PLHIV [1,8]. Also, young age groups were predominantly represented among male and transgender individuals, whereas female clients tended to be relatively older. This pattern may suggest earlier engagement of male and transgender individuals in higher-risk sexual networks and greater partner turnover.

Men constituted the majority of coinfected individuals (n = 1822, 82.5%), a pattern aligning with global evidence indicating that HIV-syphilis coinfection disproportionately affects men, particularly those engaged in high-risk sexual networks [6,8]. Surveillance data from several countries have similarly shown an increasing male-to-female ratio of syphilis cases over time [17,18]. However, much of the existing literature on HIV-syphilis coinfection focuses almost exclusively on MSM populations, reporting strong associations between coinfection and MSM behaviour [1,3,11]. However, in the present study, only 21.8% (n = 398) of coinfected males were MSM, and vaginal intercourse was the most commonly reported type of sexual act (n = 1207, 77.1%) among males, suggesting that HIV-syphilis coinfection in this population is not confined to MSM networks and may involve transmission within heterosexual and mixed sexual networks [25]. Consistent with previous literature, multivariable analysis showed that MSM status was independently associated with being unmarried, higher educational attainment, engagement in anal intercourse, and having regular and casual partners (p < 0.001) [2,20].

Female clients were significantly older (p < 0.001), less educated (p < 0.001), and reported spousal partners more frequently (n = 152, 64.7%), suggesting that transmission may often occur within stable marital relationships, where condom use tends to be low, and STI screening may occur only after symptoms appear. This pattern has been described in previous studies, where women living with HIV acquire STIs primarily through partner-mediated transmission from male spouses or regular partners, rather than through multiple partner networks [26]. In the present study, only 15% (n = 41) of female clients were identified as female sex workers, further supporting the likelihood that the majority of infections among women may have occurred within long-term or marital partnerships rather than commercial sexual encounters. In contrast, transgender individuals were predominantly younger, unmarried, and less educated (p < 0.001 each), and reported a higher frequency of anal and oral sexual practices (p < 0.001) as well as casual or commercial partners (p < 0.001). These findings are consistent with existing literature highlighting the vulnerability of transgender populations to HIV and other STIs due to structural stigma, social marginalisation, limited access to healthcare services, and engagement in high-risk sexual networks and transactional sex [27-29].

Regarding sexual behaviour patterns, vaginal intercourse was the most commonly reported type of sexual act overall (n = 1434, 76.6%); however, anal and oral intercourse were predominantly reported among transgender individuals and MSM (p < 0.001). Previous evidence suggests that anal intercourse plays a significant role in the transmission of both HIV and syphilis, since the anal mucosa is more susceptible to micro-trauma and contains a high density of target immune cells, which facilitates the transmission of sexually transmitted pathogens [2,20]. Oral sexual practices have also been identified as an underrecognized route of STI transmission, particularly in the context of inconsistent condom use [30]. Differences were also observed in the types of sexual partners reported by participants. Although spousal partners were the most frequently reported partner type overall (n = 886, 51.0%), a substantial proportion of participants also reported casual (n = 794, 44.3%) and commercial partners (n = 808, 45.9%), particularly among males and transgender individuals (p < 0.001). Among coinfected males, MSM were more likely to report casual partners, whereas non-MSM more frequently reported commercial partners and spouses (p < 0.001). These findings suggest that HIV-syphilis transmission in this cohort exists among multiple partners in marital, casual, and commercial relationships. Previous studies have reported that such relationships facilitate the creation of epidemiological “bridges,” allowing infections to spread between high-risk populations and more stable partnerships [31,32].

Condom use patterns in the present study further highlight important behavioural dynamics. Consistent condom use was highest with casual (n = 377, 49.2%) and commercial partners (n = 310, 39.5%), while it was lower with regular (n = 71, 30.6%) and spousal partners (n = 322, 39.6%). Similar patterns have been documented in other studies, where individuals often perceive stable relationships as low risk and therefore discontinue condom use [33,34]. This phenomenon has been described as risk compensation within stable partnerships, where emotional trust or perceived partner safety replaces protective behaviour despite ongoing STI transmission risk. While males had a relatively higher consistent condom use with casual partners (n = 337, 48.5%) and spouses (n = 285, 41.7%), women reported the highest consistent condom use with commercial partners (n = 39, 73.6%), likely reflecting the influence of targeted interventions and condom negotiation practices among female sex workers. However, condom use with spousal partners remained relatively low among women (n = 37, 28.9%), suggesting limited negotiation power within marital relationships [35]. Transgender individuals reported comparatively higher condom use with casual partners (n = 29, 64.4%), reflecting the targeted HIV prevention programmes implemented for key populations under the National AIDS and STD Control Programme (NACP) [27]. Condomless anal intercourse with casual partners is identified as an important behavioural factor associated with HIV-syphilis coinfection, highlighting the need for continued assessment [20,28].

The behavioural patterns should also be interpreted in the broader context of HIV awareness in India. According to the National Family Health Survey (NFHS-5) [36], although 87% of women and 94% of men aged 15-49 in India had heard of HIV/AIDS, comprehensive knowledge remained limited, with only 22% of women and 31% of men demonstrating correct knowledge of HIV prevention through consistent condom use and monogamy. In the young age group of 15-24 years, the respective proportions further dipped to 20% and 29%. Additionally, only 51% of men who reported paying for sex in the past 12 months reported using a condom during their last paid sexual intercourse [36]. These gaps in knowledge and behavioural practices may contribute to ongoing STI transmission even among populations aware of HIV.

Regarding treatment status, multivariable analysis identified treatment-naïve individuals to be generally younger, had engagement in commercial partnerships, and practised anal intercourse. On the other hand, older individuals had higher odds of being ART-experienced, likely reflecting cumulative duration of infection and sustained engagement with HIV care services. Male and transgender individuals, and individuals not currently living with a spouse, also demonstrated significantly higher odds of being ART-experienced, which may indicate better linkage to care among these groups under targeted programme interventions. These patterns may reflect behavioural modification following engagement with HIV care and counselling services. However, the persistence of inconsistent condom use across several partner categories among the ART-experienced group indicates that engagement in HIV care alone does not fully eliminate sexual risk behaviour [8,34,35].

Our study utilised a large programmatic dataset collected from 27 DSRCs across Mumbai, covering 2,209 coinfected individuals. Such use of routine programme data enhances the relevance of the findings for policy and programme implementation. However, certain limitations should be acknowledged. As the analysis was based on secondary programme data, information on several important behavioural and clinical variables was not available. Factors such as the number and gender of sexual partners, substance use, use of dating applications, partner HIV status, and clinical stage of syphilis infection were not captured in the dataset. Behavioural variables were self-reported and may therefore be subject to reporting bias or social desirability bias. In addition, the cross-sectional nature of the dataset limits the ability to establish a temporal relationship between behavioural factors and coinfection. Selection bias may exist due to the inclusion of only clinic-registered individuals. The “no response” categories in behavioural variables also demonstrated selective associations in multivariable models, particularly for casual partner status, which may reflect differential disclosure patterns, social desirability bias, or underlying unmeasured risk behaviours, and highlights the importance of cautious interpretation of self-reported behavioural data in programmatic settings. Future studies incorporating longitudinal designs and more detailed behavioural assessments would provide deeper insights into transmission dynamics.

Based on the findings of our present study, we have some recommendations. Dual HIV-syphilis testing at all HIV clinics needs to be strengthened further to enhance early detection and management of coinfection among the sexually active population. The predominance of males, young adults, and high-risk groups, particularly MSM and transgender individuals presenting with HIV-syphilis co-infection, highlights the need for targeted preventive interventions. Behavioural risk reduction counselling should be tailored according to client typology, particularly for MSM and transgender individuals who demonstrate higher engagement in anal and oral sexual practices and casual partnerships. Additionally, the persistence of high-risk behaviours such as multiple sexual partners and inconsistent condom use among ART-experienced individuals underscores the importance of regular risk assessment and positive prevention counselling during routine visits to ART centres to prevent acquisition of new STIs and onward transmission to sexual partners. Routine and periodic screening for syphilis should be ensured for all sexually active PLHIV to facilitate early diagnosis and treatment. Addressing the lower condom use with regular or spousal partners is equally critical by dispelling misconceptions about safety within trusted relationships and promoting consistent condom use even in long-term partnerships, particularly when one partner is HIV-positive. Surveillance systems can be improved further to capture behavioural risk factors, partner patterns, and reinfection rates, which would enhance understanding of STI transmission dynamics among PLHIV. Such data can further support targeted interventions and evidence-based programme planning.

## Conclusions

In our study, conducted among HIV-syphilis coinfected individuals registered for care at DSRCs in Mumbai, coinfection appears to be sustained through overlapping sexual networks involving casual, commercial, and marital partnerships combined with inconsistent condom use. This pattern highlights the complexity of HIV-syphilis transmission, where infections bridge across different partner types, and being enrolled in anti-retroviral treatment does not fully eliminate risk behaviour. Strengthening routine syphilis screening within HIV care settings, along with positive prevention counselling for behavioural risk reduction among PLHIV, is essential to interrupt these transmission networks. Emphasis on consistent condom use, regular risk assessment, and continued surveillance on behavioural risk factors can further help reduce ongoing transmission.

## References

[1] Vergara-Ortega DN, Tapia-Maltos A, Herrera-Ortíz A, García-Cisneros S, Olamendi-Portugal M, Sánchez-Alemán MÁ (2023). High prevalence of syphilis and syphilis/HIV coinfection among men who have sex with men who attend meeting places in Mexico. Pathogens.

[2] Dai W, Luo Z, Xu R (2017). Prevalence of HIV and syphilis co-infection and associated factors among non-commercial men who have sex with men attending a sexually transmitted disease clinic in Shenzhen, China. BMC Infect Dis.

[3] Mahmud S, Mohsin M, Muyeed A, Islam MM, Hossain S, Islam A (2023). Prevalence of HIV and syphilis and their co-infection among men having sex with men in Asia: A systematic review and meta-analysis. Heliyon.

[4] Wu Y, Zhu W, Sun C, Yue X, Zheng M, Fu G, Gong X (2022). Prevalence of syphilis among people living with HIV and its implication for enhanced coinfection monitoring and management in China: A meta-analysis. Front Public Health.

[5] Callegari FM, Pinto-Neto LF, Medeiros CJ, Scopel CB, Page K, Miranda AE (2014). Syphilis and HIV co-infection in patients who attend an AIDS outpatient clinic in Vitoria, Brazil. AIDS Behav.

[6] Sarigül F, Sayan M, İnan D (2019). Current status of HIV/AIDS-syphilis co-infections: a retrospective multicentre study. Cent Eur J Public Health.

[7] Mata-Marín JA, Sandoval-Sánchez JJ, Huerta-García G (2015). Prevalence of antibodies against Treponema pallidum among HIV-positive patients in a tertiary care hospital in Mexico. Int J STD AIDS.

[8] Fan L, Yu A, Zhang D, Wang Z, Ma P (2021). Consequences of HIV/syphilis co-infection on HIV viral load and immune response to antiretroviral therapy. Infect Drug Resist.

[9] Lynn WA, Lightman S (2004). Syphilis and HIV: a dangerous combination. Lancet Infect Dis.

[10] Gong HZ, Hu KR, Lyu W, Zheng HY, Zhu WG, Wan X, Li J (2020). Risk factors for the co-infection with HIV, hepatitis B and C virus in syphilis patients. Acta Derm Venereol.

[11] Su R, Liu Y, Shan D, Li P, Ge L, Li D (2025). Prevalence of HIV/syphilis co-infection among men who have sex with men in China: a systematic review and meta-analysis. BMC Public Health.

[12] Schmidt R, Carson PJ, Jansen RJ (2019). Resurgence of syphilis in the United States: an assessment of contributing factors. Infect Dis (Auckl).

[13] Spagnuolo V, Poli A, Galli L (2019). Incidence and predictors of serological treatment response in early and late syphilis among people living with HIV. Open Forum Infect Dis.

[14] Kofoed K, Gerstoft J, Mathiesen LR, Benfield T (2006). Syphilis and human immunodeficiency virus (HIV)-1 coinfection: influence on CD4 T-cell count, HIV-1 viral load, and treatment response. Sex Transm Dis.

[15] Buchacz K, Patel P, Taylor M, Kerndt PR, Byers RH, Holmberg SD, Klausner JD (2004). Syphilis increases HIV viral load and decreases CD4 cell counts in HIV-infected patients with new syphilis infections. AIDS.

[16] Zhou Y, Li D, Lu D, Ruan Y, Qi X, Gao G (2014). Prevalence of HIV and syphilis infection among men who have sex with men in China: a meta-analysis. Biomed Res Int.

[17] Read P, Fairley CK, Chow EP (2015). Increasing trends of syphilis among men who have sex with men in high income countries. Sex Health.

[18] Spornraft-Ragaller P, Schmitt J, Stephan V, Boashie U, Beissert S (2014). Characteristics and coinfection with syphilis in newly HIV-infected patients at the University Hospital Dresden 1987-2012. J Dtsch Dermatol Ges.

[19] Rekart ML, Ndifon W, Brunham RC, Dushoff J, Park SW, Rawat S, Cameron CE (2017). A double-edged sword: does highly active antiretroviral therapy contribute to syphilis incidence by impairing immunity to Treponema pallidum?. Sex Transm Infect.

[20] Zheng C, Xu JJ, Hu QH (2016). Commercial sex and risk of HIV, syphilis, and herpes simplex virus-2 among men who have sex with men in six Chinese cities. BMC Infect Dis.

[21] (2026). World Health Organization, National AIDS Control Organization. The national strategy and operational guidelines towards elimination of congenital syphilis. https://naco.mohfw.gov.in/sites/default/files/Elimination%20of%20Congenital%20Syphilis%20Book%20(2)%20(1).pdf.

[22] (2026). National AIDS Control Organization, Ministry of Health and Family Welfare, Government of India. National Guidelines for HIV Care and Treatment. https://naco.mohfw.gov.in/sites/default/files/National_Guidelines_for_HIV_Care_and_Treatment_2021.pdf.

[23] National AIDS Control Organization, Ministry of Health and Family Welfare, Government of India. (2026). National AIDS Control Organization, Ministry of Health and Family Welfare, Government of India. National technical guidelines on sexually transmitted infections and reproductive tract infections. https://naco.mohfw.gov.in/sites/default/files/National%20Technical%20Guidelines%20on%20STI%20and%20RTI_Final.pdf.

[24] (2026). National AIDS Control Organization. National Integrated Biological and Behavioural Surveillance (IBBS), India 2014-15. High risk groups. https://new.aidsdatahub.org/sites/default/files/resource/ibbs-high-risk-groups-india-2014-2015.pdf.

[25] Setia MS, Sivasubramanian M, Anand V, Row-Kavi A, Jerajani HR (2010). Married men who have sex with men: the bridge to HIV prevention in Mumbai, India. Int J Public Health.

[26] UNAIDS UNAIDS, Population Council, Saggurti N, Malviya A (2009). HIV Transmission in Intimate Partner Relationships in India. https://www.aidsdatahub.org/sites/default/files/resource/hiv-transmission-intimate-partner-relationships-india.pdf.

[27] Raj P, Dubey A (2024). Comprehending health of the transgender population in India through bibliometric analysis. Int J Public Health.

[28] Colby D, Nguyen NA, Le B (2016). HIV and syphilis prevalence among transgender women in Ho Chi Minh City, Vietnam. AIDS Behav.

[29] Storm M, Deuba K, Damas J, Shrestha U, Rawal B, Bhattarai R, Marrone G (2020). Prevalence of HIV, syphilis, and assessment of the social and structural determinants of sexual risk behaviour and health service utilisation among MSM and transgender women in Terai highway districts of Nepal: findings based on an integrated biological and behavioural surveillance survey using respondent driven sampling. BMC Infect Dis.

[30] Santa-Bárbara RC, Hueso-Montoro C, Martín-Salvador A, Álvarez-Serrano MA, Gázquez-López M, Pérez-Morente MÁ (2020). Association between sexual habits and sexually transmitted infections at a specialised centre in Granada (Spain). Int J Environ Res Public Health.

[31] Liao M, Kang D, Jiang B (2011). Bisexual behavior and infection with HIV and syphilis among men who have sex with men along the east coast of China. AIDS Patient Care STDS.

[32] Jennings JM, Tilchin C, Meza B (2020). Overlapping transmission networks of early syphilis and/or newly HIV diagnosed gay, bisexual and other men who have sex with men (MSM): opportunities for optimizing public health interventions. AIDS Behav.

[33] Cambou MC, Perez-Brumer AG, Segura ER, Salvatierra HJ, Lama JR, Sanchez J, Clark JL (2014). The risk of stable partnerships: associations between partnership characteristics and unprotected anal intercourse among men who have sex with men and transgender women recently diagnosed with HIV and/or STI in Lima, Peru. PLoS One.

[34] Santos LR, Spindola T, Hipólito RL, Barros ED, Fonte VR, Costa CM (2025). Prevention of sexually transmitted infections among young heterosexual men: a study on social representations. Rev Enferm.

[35] Marfatia YS, Naik E, Singhal P, Naswa S (2013). Profile of HIV seroconcordant/discordant couples a clinic based study at Vadodara, India. Indian J Sex Transm Dis AIDS.

[36] International Institute for Population Sciences (IIPS) and ICF (2021). National Family Health Survey (NFHS-5), 2019-21, India Report Volume 1. National Family Health Survey (NFHS-5), 2019-21, India Report Volume 1.

